# A taxonomy of impacts on clinical and translational research from community stakeholder engagement

**DOI:** 10.1111/hex.12937

**Published:** 2019-07-18

**Authors:** Sarah C. Stallings, Alaina P. Boyer, Yvonne A. Joosten, Laurie L. Novak, Al Richmond, Yolanda C. Vaughn, Consuelo H. Wilkins

**Affiliations:** ^1^ Meharry‐Vanderbilt Alliance, Vanderbilt University Medical Center Nashville Tennessee; ^2^ National Health Care for the Homeless Council Nashville Tennessee; ^3^ Department of General Internal Medicine and Public Health Vanderbilt University Medical Center Nashville Tennessee; ^4^ Department of Biomedical Informatics Vanderbilt University Medical Center Nashville Tennessee; ^5^ Community-Campus Partnerships for Health Raleigh North Carolina; ^6^ The Neighborhoods Resource Center Nashville Tennessee; ^7^ Vice President for Health Equity Vanderbilt University Medical Center Nashville Tennessee

**Keywords:** community, community-engaged research, engagement, metrics, outcome measures, patient‐centred, person‐centred, stakeholder, taxonomy

## Abstract

**Background:**

Community engagement is increasingly recognized as a valuable tool in clinical and translational research; however, the impact of engagement is not fully understood. No standard nomenclature yet exists to clearly define how research changes when community stakeholders are engaged across the research spectrum. This severely limits our ability to assess the value of community engagement in research. To address this gap, we developed a taxonomy for characterizing and classifying changes in research due to community engagement.

**Methods:**

Using an iterative process, we (a) identified areas of potential impact associated with community engagement from author experience, (b) categorized these in taxonomic bins based on research stages, (c) conducted semi‐structured interviews with researchers and community stakeholders, (d) validated the codebook in a sample dataset and (e) refined the taxonomy based on the validation. Community stakeholders were involved in every step of the process including as members of the primary study team.

**Results:**

The final taxonomy catalogues changes into eleven domains corresponding to research phases. Each domain includes 2‐4 dimensions depicting concepts within the domain's scope and, within each dimension, 2‐10 elements labelling activities through which community engagement could change research.

**Conclusions:**

Community engagement has great potential to enhance clinical and translational research. This taxonomy provides a common vocabulary and framework for understanding the impact of community engagement and suggests metrics for assessing the value of community engagement in research.

## 1 BACKGROUND

Patient and community stakeholders are being involved in re‐shaping priorities for health research, setting the research agenda, establishing a presence on proposal review committees, and translating research results into easily understood findings for the public audience.^1^, ^2^, ^3^ Viewed retrospectively, community stakeholders' contributions have added community needs to research priorities,^4^ produced culturally tailored and targeted recruitment strategies^5^ and patient‐oriented study material,^6^ enhanced approaches to research design and implementation,^7^ and improved translation and dissemination of research findings. Community Engagement (CE) Studios,^8^ focus groups, community listening sessions,^9^ advisory/oversight councils,^10^ and grant review committees are examples of strategies employed to involve community stakeholders in clinical and translational research.^11^, ^12^, ^13^ Expanding the research process to include patients, caregivers, patient advocates or members of the general public involves bringing researchers together with those who are not primarily affiliated with academic research institutions. Community stakeholder engagement, then, is multi‐disciplinary and complex, yet it lends a lived‐experience perspective so that health research itself better reflects what is most important to the population it studies and serves.^14^

Lagging behind the growth of new stakeholder engagement approaches is the development of tools for evaluating, comparing and evolving those approaches, and there is an urgent need to develop these tools to demonstrate the impact of community stakeholder engagement in research.^11^, ^15^, ^16^ Interactions between researchers and community stakeholders are not consistently captured in a standard or ordered framework, nor is the value of community stakeholders' activities to the research enterprise being measured.^17^, ^18^, ^19^, ^20^ With valuation standards and metrics, the meaningful engagement of patients and other community stakeholders could be studied scientifically and adopted with more confidence in clinical research, which is still largely done to patients as participants rather than with them as stakeholders in a bidirectional interaction.^21^, ^22^, ^23^ It is imperative to capture community stakeholder input consistently and develop measures for the value of the community stakeholder contributions to research.

There are examples in the literature demonstrating the effectiveness of taxonomies for improving metrics and scientific reporting, suggesting a taxonomy would be an effective first step in establishing a standard vocabulary and developing value measures. 24, 25, 26, 27, 28 Other stakeholder engagement efforts are illustrative of the benefits of improving vocabulary around this topic. These include the following: stakeholder engagement frameworks and guidance not focused on community stakeholders specifically,^18^, ^29^, ^30^ a scaleable approach to patient engagement for patient‐centred outcomes research (PCOR),^31^ successful patient engagement for health‐care experiences and outcomes,^32^, ^33^ and community engagement measures focused on partnership strength.^19^, ^34^ Specifically evaluating community stakeholders' contributions to research, however, needs a framework specifically focused on characterizing and measuring community representative activities through the process of conceiving, conducing, analysing and reporting clinical and translational research.

Given the complexity of community stakeholder engagement in clinical research, a taxonomy would provide a common language and framework for community stakeholder engagement that will facilitate needed standards for reporting and measures for metrics development.^35^ Over time, reporting and evaluating stakeholder engagement systematically will accelerate advancements in and adoption of community stakeholder engagement across research broadly. We developed a Community Stakeholder Impacts on Research Taxonomy to address this need.

## 2 METHODS

### 2.1 Definitions

In this work, the term “community stakeholder” includes patients, caregivers, patient advocates and members of the general public, but not payers, policy makers or health‐care product producers. A “community representative” is a person whose primary affiliation is with a non‐academic, non‐research, community‐based organization and/or who represents a defined community.^36^

### 2.2 Overview

We (a) identified areas of potential impact and outcomes associated with community stakeholder participation in clinical and translational research based on author experience, (b) categorized these in taxonomic bins based on the research cycle, (c) conducted semi‐structured interviews with researchers and community stakeholders to evaluate the resultant taxonomy, (d) validated the taxonomy in a sample dataset and (e) refined the taxonomy based on the validation. For qualitative analyses, all coding was completed using Dedoose software, an online suite for collaborative qualitative research analysis.

Our research team included leaders from two community organizations (Vaughan and Richmond) and faculty/staff from three institutions with expertise in community engagement, scale development, qualitative analysis and translational research. The experience of the team spanned facilitating CE Studios, conducting community outreach efforts, recruiting for programme participation, implementing public health interventions and evaluation, and advocating for social and economic justice. The study design, recruitment plans and semi‐structured interview questions were approved by Vanderbilt University Medical Center's IRB.

### 2.3 Identification of potential community stakeholder impacts

To generate initial content for the taxonomy, we scanned the literature reporting research in which patient, community and provider stakeholders have been involved. The content generation was guided by our team's expertise in engagement, the PCORI Patient and Family Engagement Rubric,^37^, ^38^ and a recent comprehensive review of impact.^39^ Searches in PubMed and Google Scholar included these keywords: Community‐Engaged Research (CEnR), patient and stakeholder engagement in research, participatory research, patient‐centered outcomes research, impact of community/patient/family/caregiver engagement in research, and evaluation of community/patient engagement in research.^16^, ^39^, ^40^, ^41^

### 2.4 Categorization of impacts into initial taxonomy

Two experienced faculty on our team independently reviewed the identified publications and annotated the content related to changes in research from stakeholder engagement activities. Codes were generated using an inductive approach and subsequently grouped based on thematic analysis. Through iterative rounds of review and discussion, the full team (faculty/staff and community stakeholders) developed an initial taxonomy with top‐level *domains,* representing areas where research changes might occur (ie specific research stage or overarching thematic area) and *elements,* defining the scope of activity in each domain. The elements represent activities that can be assessed or measured. We developed a codebook for qualitative analysis, making domains the parent codes and elements the subcodes.

### 2.5 Evaluation of initial taxonomy and external content collection

We conducted 12 semi‐structured interviews – six with academic researchers and six with community stakeholders – to evaluate the initial taxonomy and gather external content. One week prior to the interview, interviewees were provided with the initial taxonomy (Table 1A). Interviewees answered questions on taxonomy structure (domain nomenclature, domain arrangement and element categorizations), utility and relevance (Table 1B). Interviewees were questioned about each domain and its elements and about their overall impressions. Upon completion of the interview, both academic and community participants were compensated $50 for their time. We used a “think aloud” method to probe deeper into responses given by the interviewees to provide a richer thought process with examples.^42^ The semi‐structured interviews were recorded, transcribed verbatim and de‐identified by two research team analysts who also acted as coders. The two coders independently reviewed the transcripts and coded participants' responses to each domain and element of the taxonomy as indicating “keep”, “remove”, “add”, or “needs improvement” about that particular part of the taxonomy. Discrepancies in codes were resolved through team adjudication. Wording changes for clarity, element clustering into taxonomic dimensions, and element recategorizations were discussed among the research team to improve and refine the taxonomy in accordance with the interview results.

**Table 1:** Semi‐structured interview materials

(A) Initial taxonomy	
Potential areas of impact for patient (and other Stakeholder) engagement	
Domains	Elements
1. Pre‐research	Idea/topic generation Identify issues of greatest importance Input on Relevance/Purpose Identify stakeholders/potential partners
2. Infrastructure	Funding source decisions Preparation of budget Sharing of funds Appropriate compensation for stakeholders (patients, consumers, community organizations) Time Cost Process/structure for shared decision making
3. Research design	Define population Selection of patient‐centred tools Organize ideas and capture the way the research will be applied. Provide input on research methods Grant writing/proposal development Framing research questions Selection of comparators & outcomes Revise the research protocol Input on cultural appropriateness
4. Implementation of Research	Identify/hire research team members Recruitment of research participants Identify best approaches to recruitment and retention Determine best approaches to data collection (in person vs online vs telephone; survey vs interview; self‐report vs caregiver report) Assist with data collection
5. Analysis of Research	Assist with data analysis (train to do qualitative analysis) Provide alternative interpretation of research results (especially those that are counterintuitive) Bring attention to factors (confounders) that may not have been measured or documented in literature Interpret – assess plausibility of results Review results and provide context for relevance to patients and stakeholders
6. Dissemination of research findings	Provide culturally relevant and appropriate language Co‐authorship of manuscripts Write for non‐scientific publication Advise on appropriate audiences and non‐traditional venues for dissemination Convene town hall meetings and other opportunities for dissemination Create companion materials for dissemination – videos, newsletters, etc
7. Ethics	Consent process Acceptability of research Protection of individuals vs protection of communities Privacy (might be implied in consent process) Risks/Benefits (ie health, increased knowledge)

(B) Semi‐structured interview question		Researcher (n = 6)	Stakeholder (n = 6)
(1)	What is your first impression of the taxonomy? What makes sense to you? Are the domains/elements rational?	X	X
(2)	How would you improve the taxonomy?	X	X
(3)	Are there domains/elements you would eliminate? Why?	X	X
(4)	Would you add any domains/elements? What would you add?	X	X
(5)	Which domains/elements are you most familiar with?	X	X
(6)	Do you feel you can contribute to any of the domains/elements? If so, which ones and how?		X
(7)	Which domains/elements are most beneficial to you when seeking stakeholder input?	X	
(8)	How likely are you to provide feedback in these domains/elements?		X
(9)	Are there domains/elements that are particularly important to you?	X	X

### 2.6 Validation and refinement to final taxonomy

We piloted this revised taxonomy by asking researchers with comprehensive backgrounds in qualitative research evaluation to use the taxonomy on a sample dataset. The sample dataset was transcripts of input on research from community stakeholders in Community Engagement (CE) Studios (Joosten et al^8^) and from researchers in Translational (T2) Studios (Byrne et al, 43). CE and T2 Studios are both project‐specific consultative sessions in which individuals with expertise provide input and feedback on research. The two types of Studios are conducted similarly, but CE Studios have community stakeholders as experts while T2 Studios have researcher experts. Comparing CE and T2 Studio output affords a unique opportunity to study engagement's impact because a CE Studio is a discrete engagement method that is replicable. We used verbatim transcripts from Studio session recordings containing the input provided by community or faculty experts to investigators. Six coders not involved in developing the taxonomy were given the codebook, transcripts and a one‐hour orientation on the analysis design and objective. Analysis of studio transcripts involved reading the text, creating text excerpts and labelling each excerpt with one or more codes. Each coder received two transcripts, one from a CE Studio and on from a T2 Studio. Coder Group A (n = 3) had two transcripts with different topics (from a CE Studio on Improving Healthcare Systems and from a T2 Studio on a Chest Pain Trial). Coder Group B (n = 3) had two transcripts on the same topic, one each from a CE and T2 Studio both held on eConsent. (Table 2) Afterwards, we interviewed each coder, asking: *How did the tool work for them? What challenges did they experience? How can the taxonomy be improved? What were your overall likes and dislikes in the utility of the system?* The final taxonomy content and structure resulted from discussion in the research team. Elements describing measurable activities were binned into subdomains, or taxonomic dimensions, describing categories of research activity in each stage.

**Table 2:** Taxonomy pilot validation

		CE Studio	T2 Studio	Free codes
Coder group A. (n = 3)	Studio topic	Improving healthcare systems	Chest pain trial	Bias Buy‐in Consent Define measures Empowerment through knowledge Ethics outside of research Individualized care Layperson terms Operating in silos
	Length of transcript	40 pages	21 pages	
	# of excerpts coded	235	280	
	Primary domains (highest coding frequency)	QI, Free Codes	Research design	
Coder group B. (n = 3)	Studio topic	eConsent	eConsent	Logistics of research protocol Education of participants Tech preference Comfort level Concerns about tech access Need for clarity Role of research Tailoring to improve Language as a barrier Terminology as Barrier
	Length of transcript	36 pages	30 pages	
	# of excerpts coded	283	305	
	Primary domains (highest coding frequency)	Ethics, QI, Free codes	Ethics, QI, Free codes	

## 3 RESULTS

### 3.1 Initial taxonomy content and structure

With iteration and evaluation by the research team, 41 conceptual statements about research activities through which community stakeholder engagement is likely to be impactful were binned initially into seven top‐level taxonomic domains named for stages of research (Table 1A). The conceptual statements were products of research team members' Community‐Engaged Research (CEnR) experience as investigators, participants and community advocates for research, and the scan of related literature on Community‐Engaged Research articles (results of PubMed query run today can be found here). Example literature includes Khodyakov, et al 44, Mullins, et al^32^, and Brett, et al.^45^

### 3.2 External review results

Semi‐structured interview results from the coding of researcher and community stakeholder interview transcripts were suggestions of what in the initial taxonomy to keep, remove, add or improve. From these data and subsequent research team discussion, Translation, Quality Improvement, and Engagement domains were added and domain descriptions drafted. The amended intermediate taxonomy had 10 domains and 64 elements (not shown).

### 3.3 Pilot and validation results

Table 3 shows the numbers of codes for each taxonomic domain and the frequency difference by Studio type. Results from the qualitative analysis and the subsequent coder interviews were discussed by the research team. Based on this analysis, two domains, Communication and Post‐Research, were added.

**Table 3:** Code frequency by domain

	CE Studio	T2 Studio	Total
Validation round one			
Pre‐research	25	5	30
Infrastructure	13	2	15
Research design	74	5	79
Implementation	16	5	21
Analysis	8	10	18
Dissemination	2	17	19
Translation/post‐research	5	18	23
Ethics	35	14	49
Quality improvement	11	81	92
Engagement	1		1
Free codes	49	93	142
Validation round two			
Pre‐research	3	17	20
Infrastructure	2	2	4
Research design	8	20	28
Implementation	1	23	24
Analysis			
Dissemination		5	5
Translation/post‐research		7	7
Ethics	73	99	172
Quality improvement	82	78	160
Engagement	3	8	11
Free codes	183	206	389

### 3.4 Final taxonomy

**Figure 1:**
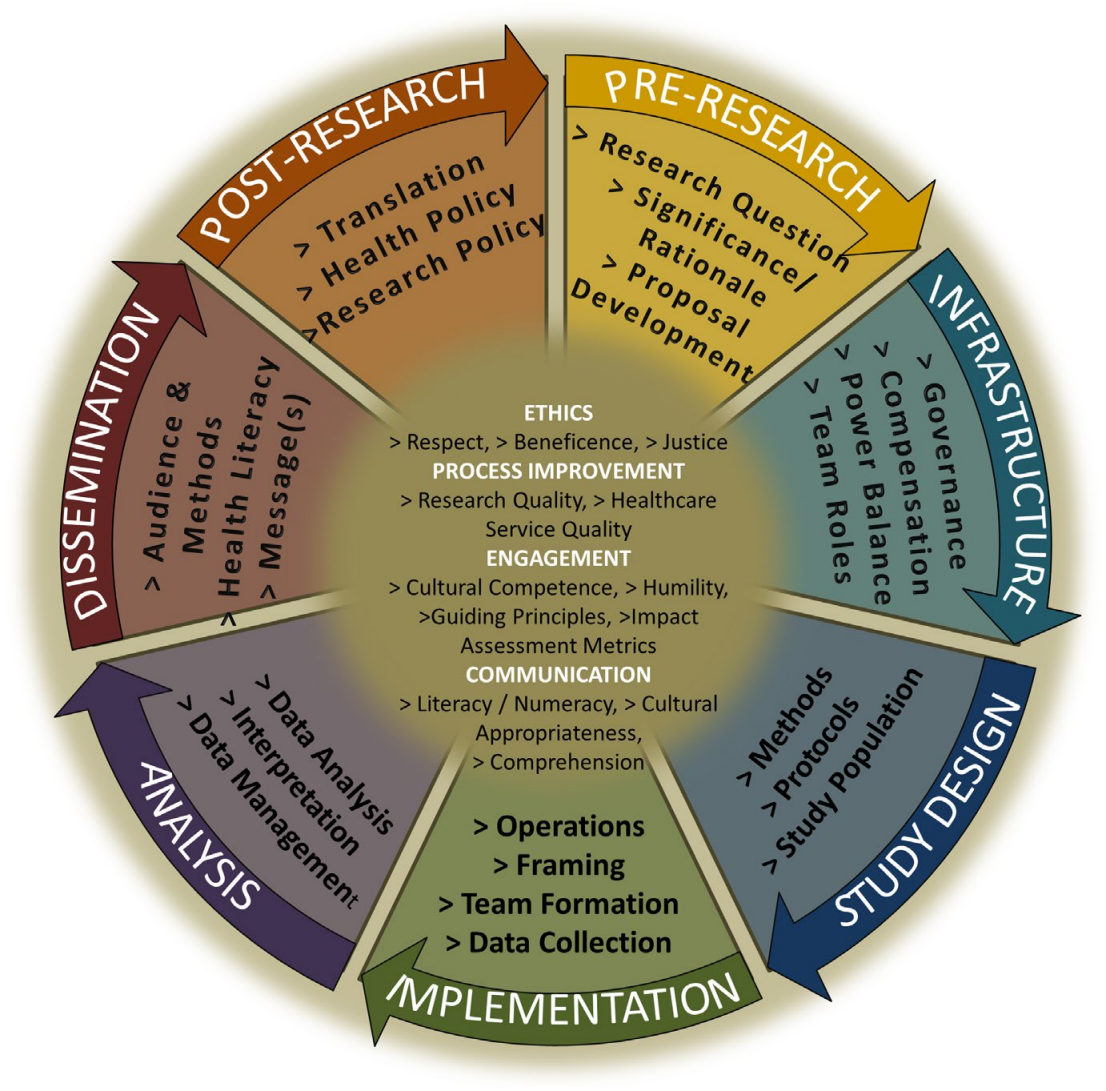
Community Engagement Impacts in Research Taxonomy: a taxonomy of standard terms for areas of community stakeholder impact in research. Domains are in all‐capital letters and white text. Dimensions (topical clusters of subcodes) are preceded by a “>” symbol and are in black text

The final taxonomy of Community Stakeholder Impacts on Research has eleven domains (codes) describing stages of clinical and translational research, 36 dimensions naming research activity concepts into which subcodes were binned, and 71 elements (subcodes) describing specific community stakeholder activities that can be assessed or measured (Table 4). Links observed while piloting the Community Stakeholder Impacts on Research Taxonomy support a cyclical and iterative model of the research process with opportunities for stakeholders to engage at all phases of research and inform next steps (Figure 1). The taxonomy systematically characterizes and categorizes community stakeholder activities that can impact the research process and also suggests possibilities for standard measures to assess that impact (Table 4 and Figure 1). Examples of possible measures are listed in the rightmost column of the taxonomy (Table 4).

**Table 4:** Taxonomy defining possible areas of impact for community stakeholder engagement in translational research

Research stages	Activity clusters	Conceptual statements about community stakeholder activities	Examples of metrics suggested by the taxonomy elements
Taxonomy domains Parent codes	Taxonomy dimensions	Taxonomy elements (Subcodes)	(CS = Community Stakeholder)
*Research stages*			
1. *Pre‐research* Stage in which the overall study and hypothesized outcomes are considered and developed.	Research question	Generate ideas Identify issues of greatest importance to community stakeholders Provide input on topic and project relevance and purpose Identify community partners Contribute to choices made in specific aims Contribute to grant writing Provide lived‐experience perspective to research question framing	# ideas generated by community stakeholders CS‐rated Importance PCoR rating of research abstract or other product Recruitment and retention rate/ improvement # and diversity of CS on team
	Significance/Rationale		
	Proposal development		
2. *Infrastructure* Stage in which logistics of the project, distribution of funds, research team members and roles, and other planning decisions are made	Governance	Add extra breadth to possible funding source lists Aid in budget preparation Consult on appropriate compensation for community research contributors (patients, consumers, community organizations), including issues around time as research team members, time for participation, and cost for travel and lost work hours. Contribute to designing a shared decision‐making process Contribute to appropriate scopes of work Contribute to decisions on participant payment system (eg what does insurance cover in a clinical study)	# of CS‐identified grant opportunities # of CS participants in grant writing process through focus groups, community engagement studios, town halls, meetings Per hour rate of CS compensation compared to other stakeholders # hours of meetings attended by CS Diversity in NIH study types with CS representation – biomedical, community engagement, cancer, etc Percent of funding that is distributed to CS or community organizations Number of educational backgrounds represented on study team $ spent to support CS participation such as virtual meeting platforms, transportation costs, reimbursements Presence or absence of a separate reimbursement structure for non‐academic participants
	Team roles		
	Balance of power		
	Compensation model		
3. *Study design* Stage of research in which how the study will be conducted, who will be included in the cohort to be studied.	Study population	Provide lived experience to the process of defining the population Provide relevant input on cultural appropriateness in the population of interest Identify potential stigmas for condition studied Provide input on the research setting and how that will impact the participants Consult on generalizability to other groups or communities Participate in selection of patient‐centred tools, including technology used during participation and data capture, literacy and numeracy levels of participant materials, clinical workflow, and impact of protocol logistics on participant experience Organize ideas and capture the way the research will be applied. Add to possible comparators and outcomes Familiarize researchers with the participants' need for clarity Assess community comfort level with study plans	Demographic diversity of participants in research study Demographic diversity in research participation overall and over time Presence or absence of systematic review for cultural appropriateness either through focus group analysis or review by health communication expert Frequency of research occurring in non‐academic settings such as churches, schools, etc Presence or absence of opportunity for CS to give feedback on study applicability to multiple study sites Range of formats for communication to participants such as phone, email, text, etc Measured PCoR score Number of modifications to research protocol made by CS Measured participant confidence in research protocol on a Likert scale
	Person‐centred methods		
	Person‐centred protocols		
4. *Implementation* Stage of research in which a research team details how the planned project is accomplished and carries out those operations	Operations	Identify possible research team members, especially among patients, caregivers, and other community stakeholders Assist with data collection Assist with participant recruitment Contribute to community needs assessment for effective and ethical consent.	Opportunity for CS involved in hiring process for research team members Presence or absence of non‐academics involved in data collection Diversity in CS responsibilities CS‐initiated suggestions for recruitment/retention that are implemented; recruitment goal achievement Presence or absence of changes to recruitment protocol after CS feedback on stigma PCoR score as related to study recruitment plan Number of participants recruited by CS Change in recruitment rate after CS input implemented Presence or absence of changes to data collection protocol after CS feedback
	Framing		
	Community‐researcher team formation		
	Data collection		
5. ***Analysis*** Stage of research during which data are analysed and interpreted	Data management	Assist with data analysis (training may be needed) Provide alternative interpretation of research results (especially those that are counterintuitive) Bring attention to factors (confounders) that may not have been measured or documented in literature Interpret and assess plausibility of results Review results and provide context for relevance to patients and their communities	Presence of training opportunities for qualitative/quantitative analysis CS participating in analysis Presence of CS authors on manuscript Presence or absence of presentations to CS to discuss analysis prior to publication
	Data analysis		
	Interpretation		
6. *Dissemination of research findings* Stage of research in which final results or intermediary outcomes or works‐in‐progress communicated orally or in writing, along with edifying impact information and requests for feedback, when appropriate. This stage can be ongoing through the project implementation	Audience & Methods	Participate in co‐authorship of manuscripts Write experience non‐scientific publication Advise on appropriate audiences and non‐traditional venues for dissemination Convene town hall meetings and other opportunities for dissemination Identify appropriate community organizations who would benefit from the research Provide input on audience for appropriate message delivery Provide advice on cultural relevance and appropriate language Participate in co‐creation companion materials for dissemination (videos, newsletters, brochures, PowerPoint presentations, handouts, etc) Conduct social media outreach Organize ideas and capture the way the research will be applied	Presence of CS coauthors # of non‐scientific publications on results # CS authors in non‐scientific publications # of presentations led by CS in non‐traditional venues; # of town hall meetings # participants in presentations at non‐traditional venues and at town halls Presence of meetings with CS to discuss results Presence or absence of review by CS for intended audience; for cultural appropriateness Number of non‐traditional media outlets identified for dissemination # of companion materials produced and reach of their distribution # of social media shares by non‐scientific organizations or individuals # of non‐scientific articles which cite the original publications
	Health/Scientific literacy		
	Culturally adapting messaging		
7. *Post‐research* Stage of research concerning translation of research findings for the purposes of improving health	Translation	Assess actionability of recommended actions, if any, from research results Suggest ways to meaningfully follow up with participants Discern overall impact of the research on the community (implications for health policy) Assist in formulating next steps, convening appropriate audiences for post‐research action Helping to formulate follow‐up research question Provide support for research in their communities (implications for research relevance and policy)	Subsequent grant funding received) Number of participants successfully contacted after study completion Ratio of investment in study expenses to that into results dissemination effort Dollars invested into research follow‐up initiatives Number of policy proposals following study completion Number of meetings held with other stakeholder, such as businesspeople, new research groups, policymakers, constituents, after publication Advocacy activity for related research in the community
	Health policy		
	Research policy		

### 3.5 Pre‐research and Infrastructure Domains

These research phases frame the overall study and the potential outcomes. Categorized in these domains are elements of the research planning process, such as proposal development and priority‐setting. The impact from involving community stakeholders here can show in a study's augmented relevance and reach. Community stakeholders can increase the patient or community centredness of the research topic, questions and hypotheses, increasing the project's relevance to the target population and possibly increasing enrollment and retention (see quote). The Governance and Policy‐making dimensions have elements reflecting how community stakeholder feedback on compensation and time/ cost burden on research participants, power balance and team roles can affect a study.

"If you want more minorities in your research, you need to change the way you tell us about your studies. Find a way to get our input, not on your terms, but ours. Put something in place for me to tell you what I want you to study?"

### 3.6 Study design and Implementation Domains

Many of the elements in these practical domains involve research logistics and operational decisions about how, where and when the research will occur. Stakeholders can provide ideas for relevant comparator groups and patient‐ or community‐centred study barriers or outcomes that may influence data collection strategies, target population decisions (see quote), recruitment materials and strategies, retention and completion methods, and best communication practices for the team.

"What do you mean by healthy? Why are you only interested in healthy people? What about including people like me who have conditions like diabetes or hypertension? We need to learn how to be healthy too. And don't you want to see how exercise can benefit people with these conditions?"

### 3.7 Analysis and Dissemination Domains

In these phases of research, the cultural relevance and appropriate language brought to interpretation and presentation affects uptake of the health message and the diversity of the research participation pool (see quote). Research and community stakeholder interview participants both described these activities benefitting greatly from patient and community stakeholders. Challenges with cross‐cultural communication that occur during these phases can be incredibly costly to research over the long term (eg the damage to research's reputation and cost to participant diversity of the poorly handled Havasupai tribe analyses in the United States^46^) and can be overcome through appropriate training between investigators and stakeholders and though peer‐to‐peer communication of results.

"I think that's where you get that openness and the willingness to really want to participate … “Well, how can I help further what you're doing? I believe in it so much, because you took the time to come and just let me ask you questions, just let me pick your brain.” … just to have that opportunity, I think, is very, very important with the dissemination. Like I said, that end right there is really the beginning of whatever you want to do, because then you have it open to you."

### 3.8 Post‐research Domain

This domain, identified during the taxonomy evaluation, centres on how research results become actions for improved health or clinical care. It was identified in an interview with a researcher during which the investigator described questions from research participants about what happens after completion of a study (see quote). The elements in the post‐research domain are activities around defining the next steps for the field, such as, *What is the next question? What type of follow‐up needs to happen now that the initial research questions were addressed? What is the overall impact on the community and what other social constructs that influence an individual's health are impacted by these results?*

"The only thing I can think of potentially, and it could be included in this, is just kind of post‐research dissemination, the follow‐up piece. … It's the biggest complaint I have received in my experience, is yeah, we participate in studies all the time and nothing happens on the back end…"

### 3.9 Ethics and Engagement Domains

The research team placed *Ethics* and *Engagement* as universal/overarching domains relevant to all phases of research and developed elements that described corresponding activities. At one point in discussion, the *Engagement* domain was divided into two subcodes reflecting the difference between community stakeholder engagement as part of the research team or research oversight board and engagement when implementing and disseminating the research findings. In the first scenario, the stakeholder role can be seen as a community advocate within research. In the second scenario, the stakeholder's role reflects advocacy in the community on behalf of research. When considering the cyclic nature of the domains and the overarching nature of the *Engagement* domain, however, it made sense to collapse those two subdomains back into one *Engagement* domain, as they simply reflect engagement activities during different research stages.

### 3.10 Process improvement Domain

In addition to providing new elements for the *Ethics* domain*,* the community interviewees identified *Process Improvement* as a domain in which they felt they had contributed guidance and oversight (see quote).

“I think a lot of times it is broken and we just don't know how to fix it because it is huge. So, someone needs to step back and go, okay, we can do this better.” … “… and I can't quote it, but I came out feeling this is really good. You know, if they can implement what we just talked about …” … “I think it really will reduce patient stress and reduce all of the risks the patients have. It doesn't make sense to be stressed out while you're in the hospital, which happens a lot. I went to the emergency room one time, and I was worse off when I left than when I went because it was stressful. So, it seems like you are cleaning up all that. And to be asked how to do that was really a good thing … just the fact that someone was looking to change it and fix it."

### 3.11 Communication Domain

*Communication*, a stakeholder impact domain that crosses all phases of research, was identified during the taxonomy pilot. *Communication* was particularly pronounced through these dimensions: Research Question, Significance/Rationale, Proposal Development, Governance (especially in the shared decision‐making activity), Person‐Centred Methods and Protocols, Interpretation, Culturally Adapting Messaging, Translation, and Policy.

## 4 DISCUSSION

The Community Stakeholder Impact on Research Taxonomy contains standardized global categories and naming structures that could be used as a defining and classifying tool, plus categorized community stakeholder activities in research that can be assessed or measured. Over time, these impact measures will build a body of comparative effectiveness knowledge crucial to both PCOR and CEnR science. The taxonomy could thereby standardize reporting and evaluation of engagement activities in research projects. Some specific examples of how the taxonomy can be used include: reporting community stakeholder engagement methods, setting criteria for patient‐centred research, and guiding those researchers seeking stakeholder input who may be unfamiliar with the possibilities for community stakeholder engagement activity. Equally, community stakeholders themselves could use the taxonomy to determine where in the process they could be most valuable in providing project‐specific input to researchers. Both scenarios add to the pragmatism of patient‐centred research. The taxonomy fills a critical gap in our ability to build an evidence base for the value of community stakeholder engagement.

Prior reports support community stakeholder engagement as an approach to increase the translation, dissemination and uptake of research findings.^45^, ^47^, ^48^ Additional evidence supports the value of community stakeholders in prioritizing research and empowering patients to be more engaged. Although community stakeholder engagement in research has been more widely embraced in recent years, literature demonstrating its value and impact is limited and is often derived from descriptive, retrospective data. Prospective studies of engagement have been case reports or qualitative analysis of engagement across multiple studies with differing types of engagement strategies and no comparison or control.^47^, ^49^, ^50^

Lacking in the literature to date are metrics and tools needed for studying community stakeholder engagement rigorously. The elements of this taxonomy, the categorized community stakeholder activities in research that can be assessed or measured, suggest possible measures that we have added to the taxonomy table to engender discussion and follow‐on research (Table 4, rightmost column). One recently developed tool to measure an outcome of engagement is the validated Person‐Centeredness of Research Scale (PCoR Scale).^51^ The PCoR Scale can be used to quantify person‐centredness in research products, and we have indicated in the taxonomy table where this scale could be effectively used to assess the impact of community stakeholder engagement.

Other community engagement logic models and frameworks guide engagement processes and allow reporting of numbers of community stakeholders and/or the strategies used mapped to different engagement goals or principles.^19^, ^31^, ^37^ Like these frames, our taxonomy contains concepts around infrastructure to support engagement (*cf*. Team Roles and Balance of Power dimensions), the value of education (*cf*. the *Analysis* domain and Health/Scientific Literacy and Translation dimensions), dissemination, expansion of research teams to include community stakeholders (*cf*. the *Infrastructure* domain), and diversity in both participants and participation activities (*cf*. the *Study Design* and *Implementation* domains). The Community Stakeholder Impact on Research Taxonomy, however, was developed from the idea of quantifying outcomes of engagement, starting with a comprehensive taxonomy vetted by community engagement academic researchers and community stakeholders. In the validation results, the difference in priorities and even feedback styles of the community stakeholder and the academic faculty Studio experts are reflected in the distinctively different code frequencies observed in analysis. The method we used is reproducible, allowing for building in new concepts as community stakeholders engagement increases and is evaluated through the taxonomy. Further, we learned from our interviews that community stakeholders often want to follow up about study results, support the research through advocacy in their community, continue to be involved through informing follow‐up research questions, and participate in the research itself. That the conceptual elements used to build the taxonomy came from researchers and community stakeholders supports our view that collaborative stakeholder involvement, rather than consultative involvement only, favours full and continued engagement. The potential of the Community Stakeholder Impacts on Research Taxonomy to guide community engagement reporting standards and metrics development supports its adoption and use and indicates its implications for engagement science.

### 4.1 Limitations

The listing of measurable elements can and will grow as we were not able to capture every existing encounter between researchers and stakeholders. This taxonomy was pilot tested on transcripts from real‐world studios; however, this does not capture all contexts in which stakeholders are engaged. This limitation is reflected in the high number of free codes found during the validation (Table 3). Since the method we used is reproducible and the taxonomy flexible, new concepts can be built in as different engagement contexts are evaluated using the taxonomy.

The taxonomy development process revealed cross‐over concepts. Some conceptual elements uncovered in our study belong in more than one dimension and even more than one domain. We believe, however, that this mirrors the research process itself, which is iterative and not always linear. The cross‐over elements also reflect the complexity of investigator—community stakeholder interactions. Many research activities repeat, iterate and occur in multiple process domains. For example, stakeholders can share input on creating materials (such as recruitment materials), survey design and summary of results, activities which can occur in the Research Design, Implementation, and/or Dissemination domains. This multiplex hierarchical structure is common in medical terminology and similar to that seen in medical subject headings (MeSH). The taxonomy's illustration of, and standard structure for, areas of value from stakeholder input is its primary contribution.

## 5 CONCLUSIONS

Community engagement has great potential to enhance clinical and translational research. The Community Stakeholder Impact on Research Taxonomy provides a common vocabulary and framework for understanding the impact of community engagement and suggests metrics for assessing the value of community engagement in clinical and translational research. The taxonomy organizes the complexity of engagement into a framework that can be used to consistently report engagement activities and measure their impact. Measuring stakeholder impact as engagement strategies are envisioned and tried will drive increased stakeholder involvement and channel it towards the most effective strategies, a needed advance for this field. We anticipate types of engagement will grow as engagement science grows. We see value in the taxonomy's flexibility, and in the reproducibility of the method used to devise it, to capture that growth in a structured way.

### 5.1 Ethics approval and consent to participate

The reported approach and interview questions were approved by Vanderbilt University's IRB (#140955). The study was deemed exempt, and the consent procedures were approved. For the randomization to CE or T2 Studios part of the study, investigators were administered a survey and survey completion served as implied consent and was recorded with the survey responses in REDCap. The following is the consent statement at the beginning of each survey:

You have been asked to complete this survey because of your participation the Community Engagement Studio. The main benefit to completing this survey is to improve the effectiveness of the Community Engagement Studio, and to understand the impact it has on research. Your individual responses may be included in a research study and will be anonymous. There are no known risks to completing this survey, and your participation is voluntary. Refusing to participate will not have any impact on your access to a Community Engagement Studio in the future.

For the structured interviews, consent was granted verbally and recorded along with the interview. The following is the consent statement at the beginning of each structured interview:

In order to improve the use and understanding of the instrument, I will be asking you a series of questions to better understand your perception and understanding of its use. Please be open and honest with your responses. This interview will be audio‐recorded. If you would like to stop the interview at any point in time, feel free to do so. Audiotapes will not be released to the public. Do you agree to participate in this interview and to audiotaping? [Confirm agreement before continuing.]

## CONFLICT OF INTEREST

The authors declare that they have no competing interests.

## DATA ACCESIBILITY

Data sharing is not applicable to this article as no new data were created or analysed in this study.
